# *Plasmodium berghei* serine repeat antigen 3 (PbSERA3) is required for hepatic merozoite egress

**DOI:** 10.1128/mbio.03818-25

**Published:** 2026-01-30

**Authors:** Dipti Singh, Smita Patri, Narahari Veeda, Chandan Kumar Verma, Anusha Kavati, Rameswara R. Segireddy, Surendra Kumar Kolli, Kota Arun Kumar

**Affiliations:** 1 Department of Animal Biology, School of Life Sciences, University of Hyderabad 28614, Hyderabad, Telangana, India; Rutgers-New Jersey Medical School, Newark, New Jersey, USA

**Keywords:** *Plasmodium berghei*, serine repeat antigens (SERA), sporozoite, exo-erythrocytic forms, egress, proteolytic processing

## Abstract

The asexual replicating intracellular stages of *Plasmodium* reside within the parasitophorous vacuole (PV) and become extracellular through an active, protease-mediated process known as egress. While egress is critical for parasite viability and dissemination, the number of effector proteases involved in this process remains unknown. The genome of *P. berghei*, a rodent malaria parasite, encodes five serine repeat antigens (SERAs) that bear a central papain protease domain and a serine or cysteine residue in their active site. Of these, only *Pbsera3* is indispensable for blood-stage development, thus limiting its functional investigation. Using a yeast-based Flp/*FRT* conditional mutagenesis system, we successfully obtained *Pbsera3*-edited sporozoites that invaded hepatocytes and developed normally in late liver stages but failed to initiate a blood-stage infection. Interestingly, we observed PbSERA3 localization on the surface of ookinetes and sporozoites under non-permeabilized conditions, likely indicating its extracellular nature. Further, PbSERA3 is processed in ookinete and sporozoite stages, yielding two bona fide products as reported in the blood stages. Given that PbSERA3 and its ortholog in *P. falciparum* (PfSERA6) are substrates for subtilisin-like protease (SUB1), proteolytic maturation of PfSERA6 may also be a conserved and critical event for hepatic egress. Developing inhibitors that prevent maturation of PbSERA3/PfSERA6 may have important therapeutic implications for preventing clinical malaria.

**IMPORTANCE:**

The intracellular stages of *Plasmodium* that replicate asexually reside within a vacuole delimited by a parasitophorous vacuolar membrane (PVM). A family of serine-rich antigens (SERAs), with a cysteine residue in its catalytic site, is implicated in liberating these parasites from PVM. In *P. berghei*, a rodent malaria parasite, PbSERA3, an ortholog of PfSERA6, is indispensable for the parasite. However, its maturation by another parasite protease called subtilisin 1 (SUB1) is critical for its effector functions. During EEF development, the processed PbSERA3 is translocated across the PVM and possibly implicated in hepatic takeover. A direct role of PbSERA3 in liver stages is lacking to date. Our study generated conditional mutants of PbSERA3 and demonstrated normal development of the mutant in hepatocytes, but an inability to cause blood-stage infection. These observations point to the role of PbSERA3 in hepatic egress. We further demonstrated the extracellular nature of PbSERA3 in the ookinete, midgut, and salivary gland sporozoite stages, with a bona fide processing pattern similar to that of blood stages. Our studies demonstrated the essentiality of PbSERA3 in liver stages, making it an attractive target for antimalarial therapy. As PbSERA3 mutants manifest a late developmental arrest in the liver, they have implications in eliciting cross-stage immunity, owing to a shared repertoire of antigens with blood stages.

## OBSERVATION

Egress of *Plasmodium* parasites from the host cell across multiple life cycle stages facilitates the dissemination and continuation of the life cycle. Cysteine protease inhibitors prevent the proteolytic maturation of parasite substrates, affecting the egress of hepatic (1) and erythrocytic stages (2, 3), suggesting that this is a protease-dependent mechanism. Intra-hepatic and erythrocytic stages are delimited by PVM (4), whose rupture is an active process mediated by a family of papain-like proteases referred to as “serine-repeat antigens” (SERAs) (5). SERAs are classified into serine or cysteine type based on the active site residues (5). *P. falciparum* encodes nine putative cysteine proteases, of which eight SERA-coding genes (*Pfsera1-8*) reside on chromosome 2 (6), while the *Pfsera9* is located on chromosome 9 (7). Among the PfSERAs, PfSERA5 and PfSERA6 are indispensable for erythrocytic stages (8, 9), are secreted in PV (10, 11), and are cleaved by a serine protease, called subtilisin 1 (PfSUB1) (11, 12). Although both PfSERA5 and PfSERA6 regulate parasite egress from RBC, PfSERA5 lacks the conventional protease domain and is therefore classified as a pseudogene (13). *P. berghei* has five SERA-coding genes (5) organized tandemly on chromosome 3. In *P. berghei*, all SERAs are functionally well-characterized, except PbSERA3, an ortholog of PfSERA6. For example, PbSERA1 and PbSERA2 are expressed in mature schizonts and in late liver stages (14). In EEFs, PbSERA1 localizes to PVM, while PbSERA2 is associated with hepatic merozoites (14). However, their depletion did not affect parasite viability, although a compensatory induction of PbSERA3 was observed (14). PbSERA4 is expressed and proteolytically processed in both blood and liver stages and is required for normal asexual propagation and liver egress, consistent with its localization in hepatic merozoites (15). PbSERA5, also called ECP1 (16), is required for normal sporozoite egress from oocyst, and mutant sporozoites that lack PbSERA5 experience a defect in CSP processing, an essential step in sporozoite maturation, resulting in their inability to exit oocyst.

PbSERA3 is expressed in blood and liver stages (17), and in hepatic stages, it is processed by PbSUB1 to produce two bona fide products of 72 and 55 kDa (18). The PfSERA6, an ortholog of PbSERA3, is processed by PfSUB1. In *P. berghei,* lack of processing of SERA3 in the absence of SUB1 resulted in the failure of PVM rupture in hepatic stages. However, in *P. falciparum*, depletion of SERA6 did not compromise PVM rupture but impacted RBC membrane rupture, thereby preventing egress (19). Whether PbSERA3 also plays a role in the egress of liver stages is not known. Interestingly, PbSERA3 maturation by SUB1 also occurs in the gametocyte stage before its activation (20). PbSERA3 is transcriptionally active during late liver stages. During the cytomere stage, it localizes to the parasite cytoplasm and PVM, with traces in the PV. Interestingly, the processed forms of PbSERA3 are also detected in hepatocyte cytoplasm (17). The export of processed PbSERA3 into the host cytosol is speculated to contribute to the detachment and death of host cells (17). However, due to the essentiality of the *Pbsera3* locus (17), its role in liver stages remains unexplored. The current study addresses this lacuna and demonstrates the role of PbSERA3 in the liver stage egress by using a conditional mutagenesis system.

**Fig 1:**
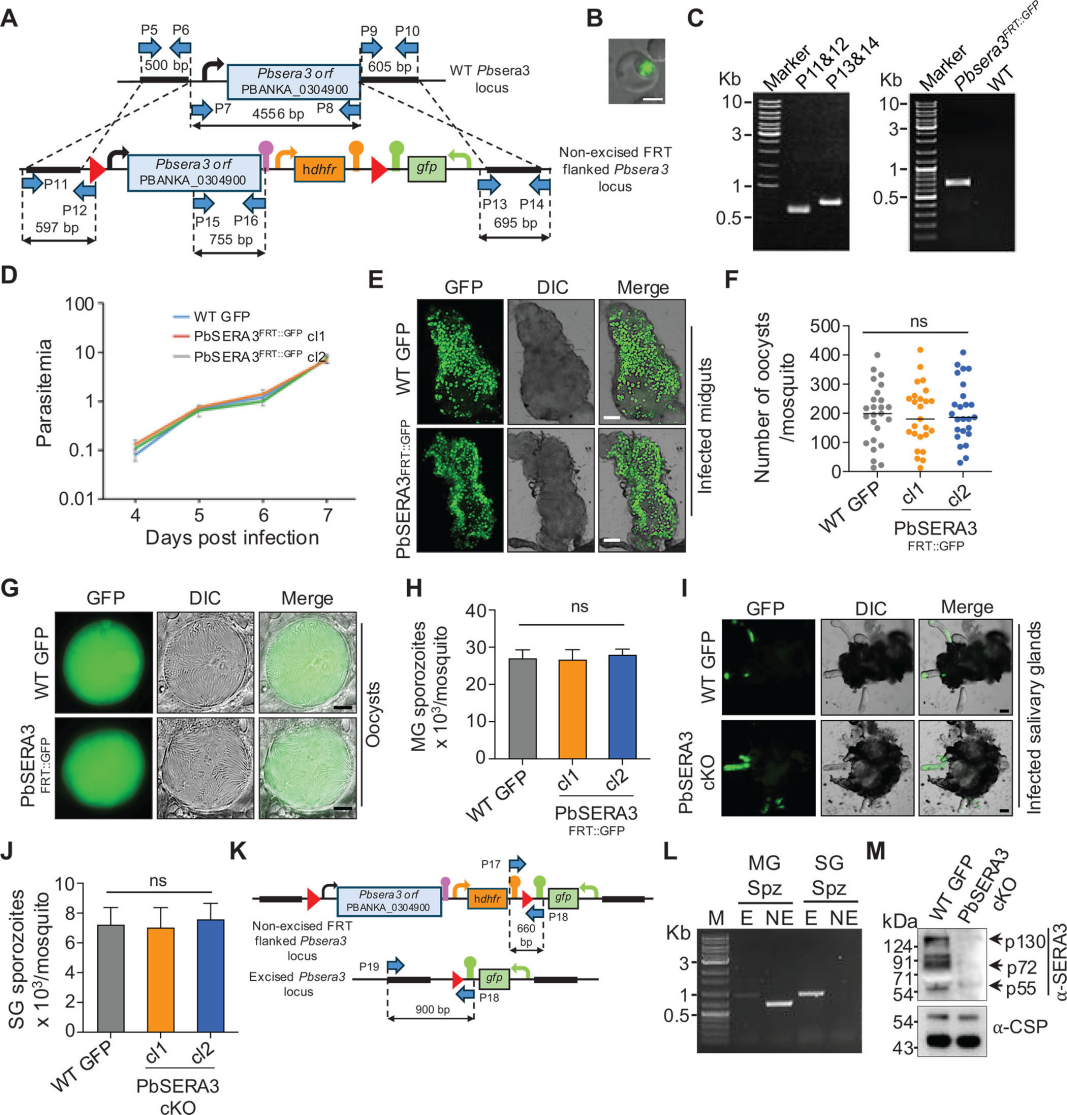
Generation and phenotypic analysis of PbSERA3^FRT::GFP^ line in asexual stage and mosquito stages. (**A**) Schematic showing a strategy employed for generating the Pbsera3^FRT::GFP^ line. The top panel shows *Pbsera3* wild-type (WT) locus. The lower panel shows an engineered *Pbsera3* promoter and *orf* with an upstream *FRT* site, a downstream h*dhfr* cassette, a second *FRT*, and a *gfp* cassette. The *FRT* sites are shown as red triangles. Thick lines indicate the regions used for homologous recombination. Blue arrows indicate the location of primers. (**B**) RBC harboring PbSERA3^FRT::GFP^ parasite (scale bar: 3 μm). (**C**) Diagnostic PCR products with indicated primer sets confirming correct integration of the engineered construct in the *Pbsera3* locus (left panel) and the confirmation of cloned PbSERA3^FRT::GFP^ line by diagnostic PCR using primers P15 and P16 (right panel). (**D**) Line graph showing the asexual propagation of WT GFP line and two independent PbSERA3^FRT::GFP^ clones (cl1 and cl2). Data were obtained from three independent experiments. (**E**) Infected mosquito midguts showing oocysts on day 14 from WT GFP and PbSERA3^FRT::GFP^ lines. Scale bar: 200 μm. DIC, differential interference contrast. (**F**) Dot plot showing day 14 oocyst burden (*n* = 25). Horizontal line represents median; ns, not significant. (**G**) Sporulation pattern inside oocysts derived from WT GFP and PbSERA3^FRT::GFP^. Scale bar: 20 μm. (**H**) Bar graph showing quantification of midgut (MG) sporozoite numbers on day 14. Data were obtained from three independent experiments, each with at least 25 mosquitoes per experiment. Each bar represents the mean with standard deviation; ns, not significant. (**I**) Dissected mosquito salivary glands (SG) harboring GFP-expressing WT and PbSERA3 cKO sporozoites. Scale bar: 200 μm. (**J**) Bar graph showing salivary gland sporozoite load on day 20–21. Data were obtained from three independent experiments, each with at least 50 mosquitoes. Each bar represents the mean with standard deviation; ns, not significant. (**K**) The top and bottom panels show the non-excised and excised PbSERA3 ^FRT::GFP^ engineered locus, respectively. *FRT* sites are shown as red triangles. Blue arrows indicate the location of primers used to confirm excised and non-excised loci. (**L**) Agarose gel image showing diagnostic PCRs performed from genomic DNA to detect PbSERA3 excised (**E**) and non-excised (NE) population from midgut (MG) and salivary gland (SG) sporozoites. The PCR products indicate the presence of maximal PbSERA3 non-excised population in MG and excised population in SG. (**M**) Western blotting showing the depletion of PbSERA3 in the lysates of PbSERA3 cKO sporozoites. Anti-CSP monoclonal antibody is used as a loading control.

To generate a PbSERA3 conditional knockout, we utilized a yeast-based Flp/*FRT* conditional system. Generation of this line involved the replacement of *Pbsera3* locus with a targeting construct carrying engineered *Pbsera3*, flanked by *FRT* (Flp Recognition Target) sites and a GFP cassette. The engineered construct was transfected into an flp recombinase-expressing parental line (21) (Fig. 1A). Parasite transfection was performed following the standard procedure, and recombinants were enriched under pyrimethamine selection (22). The recombinant parasite line expressing GFP was referred to as PbSERA3^FRT::GFP^ (Fig. 1B), and its genotype was confirmed by 5′ and 3′ integration PCRs (Fig. 1C, left panel). Following limiting dilution, a clonal line of the recombinant parasite was established and confirmed by diagnostic PCRs (Fig. 1C, right panel). The recombined locus was further confirmed for the presence of *FRT* sites by Sanger sequencing (data not shown). For the phenotypic characterization of PbSERA3^FRT::GFP^ line, two clones obtained from independent transfections were used. Analysis of asexual propagation revealed that PbSERA3^FRT::GFP^ replicated at similar rates to the WT GFP line (23) (Fig. 1D). Upon transmission to mosquitoes, the PbSERA3^FRT::GFP^ line produced a similar number of oocysts (Fig. 1E and F), with a normal sporulation pattern (Fig. 1G) and midgut (MG) sporozoite load comparable to WT GFP parasites (Fig. 1H). To activate Flp recombinase for the excision of the *FRT*ed *Pbsera3* locus, mosquitoes harboring the PbSERA3^FRT::GFP^ parasites were maintained at 25°C from days 16 to 21 post-blood meal. On day 21, we observed that the PbSERA3^FRT::GFP^ sporozoites successfully colonized the salivary glands (SG) (Fig. 1I), and their load was comparable to that of WT GFP (Fig. 1J). A scheme for diagnostic PCR to detect the excised and non-excised *Pbsera3* locus in the sporozoite population is shown in Fig. 1K. Consistent with the expression of FLP recombinase in the oocyst stage, diagnostic PCR from the genomic DNA detected the onset of excision on day 16 MG sporozoites (Fig. 1L). The excision was nearly complete in SG sporozoites on day 21 (Fig. 1L), producing PbSERA3 conditional knockout parasites (PbSERA3 cKO). Concomitant with this observation, we noted depletion of PbSERA3 protein in lysates prepared from conditionally silenced sporozoites (Fig. 1M).

**Fig 2:**
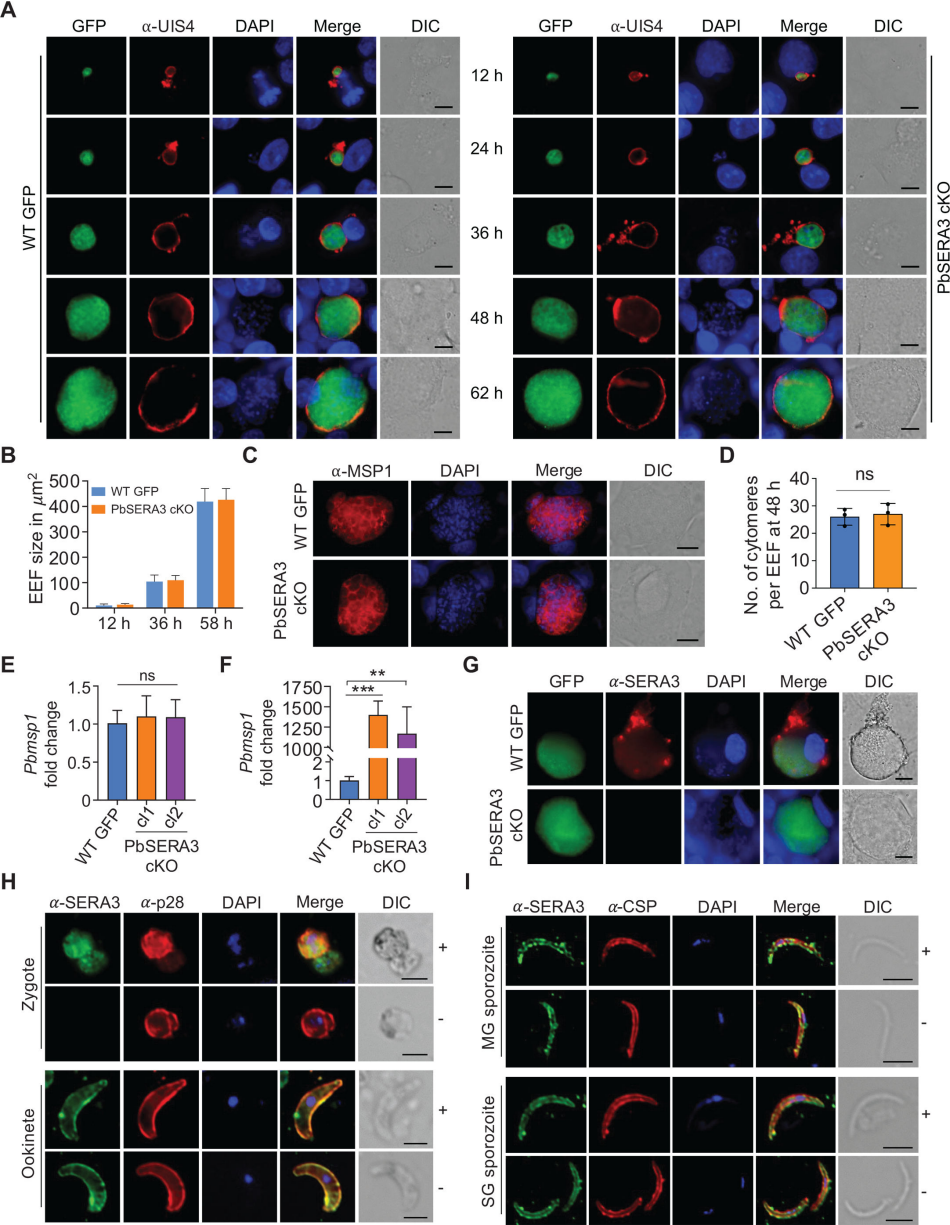
Phenotypic characterization of PbSERA3 cKO in liver stages and PbSERA3 localization in sexual and sporozoite stages. (**A**) EEF development of GFP-expressing WT and PbSERA3 cKO mutants in HepG2 cells at the indicated time points. The parasitophorous vacuolar membrane (PVM) was stained with rabbit anti-UIS4 antibody and revealed with Alexa Fluor 594-conjugated anti-rabbit secondary antibody. The host and parasite nuclei were stained with DAPI (scale bar: 10 μm). (**B**) Bar graph showing area of EEFs from WT GFP and PbSERA3 cKO at the indicated time points. Each bar represents the mean EEF area with standard deviation (*n* = 20). (**C**) Fluorescent images showing the staining of cytomeres at 48 h with anti-PbMSP1 mouse monoclonal antibody and revealed with Alexa Fluor 594-conjugated anti-mouse secondary antibody. The host and parasite nuclei were stained with DAPI (scale bar: 10 μm). (**D**) Bar graph showing quantification of WT GFP and PbSERA3 cKO cytomeres at 48 h. The data were obtained from three independent experiments, each with 25 EEFs. Each bar represents the mean with standard deviation. ns, not significant. (**E and F**) Quantitative real-time PCR showing the *Pbmsp1* expression normalized with mouse *gapdh* following 2^-△△Ct^ method at 48 h (**E**) and 70 h (**F**) from WT GFP and two independent clones (cl1 and cl2) of PbSERA3 cKO. Statistical differences were determined by one-way ANOVA with Tukey’s multiple comparisons test. ns, not significant, **P = 0.0013 and ***P = 0.0005. (**G**) Fluorescent images showing the absence of PbSERA3 staining in 65 h EEFs derived from PbSERA3 cKO sporozoites. PbSERA3 was revealed using rabbit anti-PbSERA3 polyclonal antibody, and immunoreactivity was revealed using Alexa Fluor 594-conjugated anti-rabbit secondary antibody (scale bar: 10 μm). (**H and I**) Localization of PbSERA3 in zygote, ookinete, MG, and SG sporozoites following staining with rabbit anti-PbSERA3 polyclonal antibody under permeabilized (+) and non-permeabilized (−) conditions. Anti-p28 was used as a marker for zygote and ookinete. Anti-CSP antibody was used as a marker for MG and SG sporozoites. Parasite nuclei were stained with DAPI (scale bar: 5 μm).

PbSERA3 cKO sporozoites were further analyzed for their growth in hepatocytes. In four independent experiments, 5 × 10^3^, 1 × 10^4^, 5 × 10^4^, and 1 × 10^5^ PbSERA3 cKO sporozoites were intravenously injected into mice (*n* = 5 or 3). As a control, mice received 5 × 10^3^ and 1 × 10^4^ WT sporozoites (*n* = 5). Pre-patency was monitored in all groups of infected mice using Giemsa-stained tail blood smears. Mice infected with PbSERA3 cKO sporozoites did not initiate blood-stage infection, irrespective of the dose (Table 1). To test whether lack of PbSERA3 results in developmental arrest of EEF, 2 × 10^4^ PbSERA3 cKO sporozoites were added to HepG2 monolayers, and *in vitro* EEF development was monitored at 12, 24, 36, 48, and 62 h post-infection by staining with anti-UIS4 (24), a PVM marker. Additionally, 48 h EEFs were also stained with anti-MSP-1 to analyze and quantify cytomere formation. The growth (Fig. 2A) and size (Fig. 2B) of mutant parasites were comparable to WT at all time points, including the formation of cytomeres (Fig. 2C and D). The lack of egress in PbSERA3 mutants was further demonstrated *in vivo* by exposing groups of mice to 1 × 10^4^ *PbSERA3* cKO or WT sporozoites, followed by quantification of PbMSP1 levels in infected livers. The relative fold change in levels of *P. berghei msp1* showed no difference in parasite load at 48 h (Fig. 2E), mirroring *in vitro* EEF development studies. However, at 70 h, the mutants showed a higher relative fold change in *Pbmsp1* compared to the WT (Fig. 2F), reiterating an egress-defective phenotype. To demonstrate that lack of egress was a consequence of PbSERA3 depletion, we stained 65 h *in vitro* EEFs with anti-PbSERA3 antibody. We noted that the conditional mutants failed to produce signals with anti-PbSERA3 antisera, while WT EEFs expressed PbSERA3 prior to hepatic egress (Fig. 2G). Staining 65 h EEFs with pre-immune serum did not show any fluorescent signal, thus confirming the specificity of PbSERA3 antiserum (Fig. S1). Taken together, the *in vitro* and *in vivo* studies suggest a role for PbSERA3 in initiating blood-stage infection by regulating parasite egress from hepatocytes.

**TABLE 1:** the pdfPre-patency in C57BL/6 mice exposed to different doses of PbWT GFP and PbSERA3 cKO sporozoites

Expt. No.	Parasite strain	No. of sporozoites injected (i.v.)	No. of mice used/No. of mice positive for blood-stage infection	Pre-patency period (in days)
1	WT GFP	5 × 10^3^	5/5	3.5
	PbSERA3 cKO	5 × 10^3^	0/5	None
2	WT GFP	1 × 10^4^	5/5	3.5
	PbSERA3 cKO	1 × 10^4^	0/5	None
		5 × 10^4^	0/5	None
		1 × 10^5^	0/3	None

As localization studies for PbSERA3 exist only for the hepatic and erythrocytic stages, we were intrigued to analyze the cellular distribution of PbSERA3 in mosquito stages, such as the zygote, ookinete, MG, and SG sporozoites. To this end, we employed different *in silico* tools to predict functional motifs in PbSERA3. A Phobius posterior probability and TMHMM score of nearly 1 informed a non-cytoplasmic, extracellular nature of the PbSERA3, together with predictions of the signal peptide (SP) and short transmembrane (TM) helix (Fig. S2A and B). HMMTOP predicted TM helix between aa 224–248 (Fig. S2C). SignalP-5.0 and Deeploc-2.0 predicted the SP and its cleavage site between amino acid 21 (ser) and 22 (asp) (Fig S2D, S3A and B). To validate the *in silico* predictions, we stained the sexual stages (zygote and ookinete) (Fig. 2H) and sporozoites (MG and SG) (Fig.2I) under both permeabilized and non-permeabilized conditions with the anti-PbSERA3 antibody. Interestingly, we detected PbSERA3 in the cytoplasmic compartment of zygotes. However, in ookinetes, MG, and SG sporozoites, we noted PbSERA3 signals also in non-permeabilized conditions, inferring an extracellular association with the membrane. Tubulin was used as an intracellular marker to validate the permeabilization conditions (Fig. S4). This concurred with predictions of Phobius and TMHHM. Further, it is likely that in extracellular stages, the signal sequence sorts PbSERA3 into the secretory pathway. Upon membrane fusion, PbSERA3 remains extracellular, except at its predicted transmembrane helix region (aa 224–248). The PbSERA3 immunoreactivity on the membranes of ookinete, MG, and SG sporozoite stages colocalized, respectively, with p28 and CSP, the membrane markers for ookinetes and sporozoites. The colocalization was measured by Pearson’s correlation coefficient, and its values ranged from 0.78 to 0.89 (Fig. S5). The antiserum was also used to study the processing of PbSERA3 in the ookinete and sporozoite stages, which revealed a similar pattern as noted in the blood stages (Fig. S6) (17).

Previous studies have shown that processed forms of PbSERA3 translocate into the hepatocyte cytoplasm around 56 h post-infection, a process that is inhibited by E-64 (17). Though implicated in host cell takeover, a precise role of PbSERA3 in hepatocyte modulation is lacking due to the indispensable nature of the *Pbsera3* locus (17). By a conditional mutagenesis approach, we demonstrated a role of PbSERA3 in hepatic egress, mirroring the chemical inhibition achieved by E-64. A likely explanation for a similar outcome by two independent approaches points to the possibility that hepatic takeover (17) is compromised, resulting in the failure to produce merosomes (1). The molecular mechanisms that activate the downstream effectors of processed PbSERA3 in egress need further investigation. Previous studies have shown that conditional depletion of PbSUB1 results in a lack of PbSERA3 processing and a defect in hepatic egress (18). Surprisingly, PbSERA3 mutants also mimic the phenotype of PbSUB1 mutants. Taken together, both these studies reiterate that hepatic takeover and consequent egress are common outcomes following depletion of PbSERA3 or its maturase, PbSUB1. The current study, therefore, fills an important gap in understanding the role of PbSERA3 in the egress of liver stages.

Whether PbSERA3 processing in the ookinete and SG sporozoite stages has any functional implications for the parasite is not known. In fact, in *P. berghei*, the SERA3 processing governs several stage-specific functions. For example, in erythrocytic stages, PbSERA3 and its Pf ortholog acquire autoprotease activity following SUB1-mediated processing, an event critically dependent on conserved cysteines (11), which mediates egress. PbSUB1 also processes PbSERA3 upon male gametocyte activation, facilitating male gamete egress (20). Maturation of PbSERA3 also occurs in liver stages as described above (17). Whether the ookinete membrane association of PbSERA3 has proteolytic functions needs further validation. However, some studies demonstrated the expression of certain aspartic proteases, such as an ortholog of PfPM4 (25) and PM VII and X (26) in ookinetes. It may be possible that these proteases may act in concert, leading to the disruption of the mosquito gut barrier or inactivating immune effectors prior to oocyst formation. Interestingly, PbSERA3 depletion did not affect EEF development, suggesting that mutant sporozoites were able to invade hepatocytes normally. A likely explanation is that PbSERA3 produced in the MG sporozoite stage, before the onset of excision of the *Pbsera3* locus, may suffice for sporozoite invasion of hepatocytes. Such a prediction is based on the IFA studies in MG sporozoites (Fig. 2I).

### Conclusion

The current study employed an elegant conditional depletion approach to silence the expression of PbSERA3 in the pre-erythrocytic stages and demonstrated its role in hepatic egress. Thus, chemical inhibition of PbSERA3 or its depletion appears to be a promising approach for preventing clinical malaria. Chemical intervention in PbSERA3 processing may affect multiple life cycle stages, yielding a more holistic approach to controlling malaria. Further, the mutants possibly can induce cross-stage immunity, owing to a late liver stage arrest, which has a repertoire of antigens shared with blood stages. Taken together, PbSERA3 and its Pf orthologs are promising targets for developing antimalarials and late liver-arresting genetically attenuated parasites that can be used as a whole-organism vaccine.

## Data Availability

All data supporting the findings of this study are available within the manuscript and its Supplementary Information.

## SUPPLEMENTAL MATERIAL

Supplemental material mbio.03818-25-s0001.pdf Figures S1 to S7, supplemental methods, and Table S1.

ASM does not own the copyrights to Supplemental Material that may be linked to, or accessed through, an article. The authors have granted ASM a non-exclusive, world-wide license to publish the Supplemental Material files. Please contact the corresponding author directly for reuse.
