# Supplementary material for: Plasmodium berghei serine repeat antigen 3 (PbSERA3) is required for hepatic merozoite egress
Source: mBio. 2026 Jan 30;17(3):e03818-25. doi: 10.1128/mbio.03818-25 (PMC12977625; doi:10.1128/mbio.03818-25)
Supplement: Supplemental material — Figures S1 to S7, supplemental methods, and Table S1. [file mbio.03818-25-s0001.pdf]

Supplementary material for

Plasmodium berghei Serine Repeat Antigen 3 (PbSERA3) is required for hepatic merozoite egress

Dipti Singh[#], Smita Patri[#], Narahari Veeda, Chandan Kumar Verma, Anusha Kavati, Rameswara R Segireddy, Surendra Kumar Kolli* and Kota Arun Kumar*

Department of Animal Biology, School of Life Sciences, University of Hyderabad, Hyderabad, Telangana, 500046, India
# These authors contributed equally.
*Corresponding Authors: Kota Arun Kumar (kaksl@uohyd.ac.in),
Surendra Kumar Kolli (skk@uohyd.ac.in)

This file contains

Supplementary figures **S1 to S7**

Supplementary information - **Materials and Methods**
- **References**

Supplementary Table **S1 - List of Primers**

Figure S1

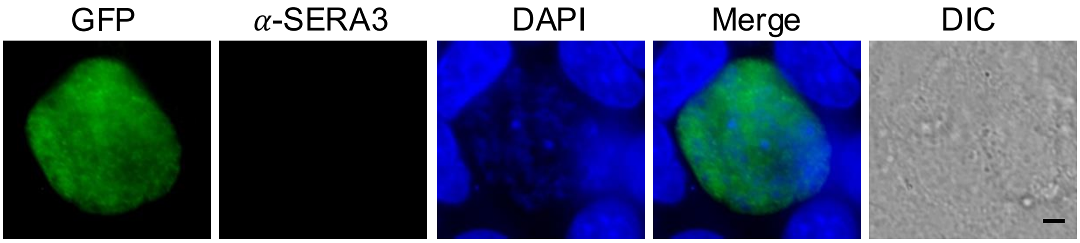

Figure S1: Fluorescent microscope image of 65 h WT GFP EEF stained with pre-immune rabbit serum and revealed with Alexa Fluor 594 conjugated anti-rabbit secondary antibody. Nuclei were stained with DAPI (Scale bar - 10 μm).

Figure S2

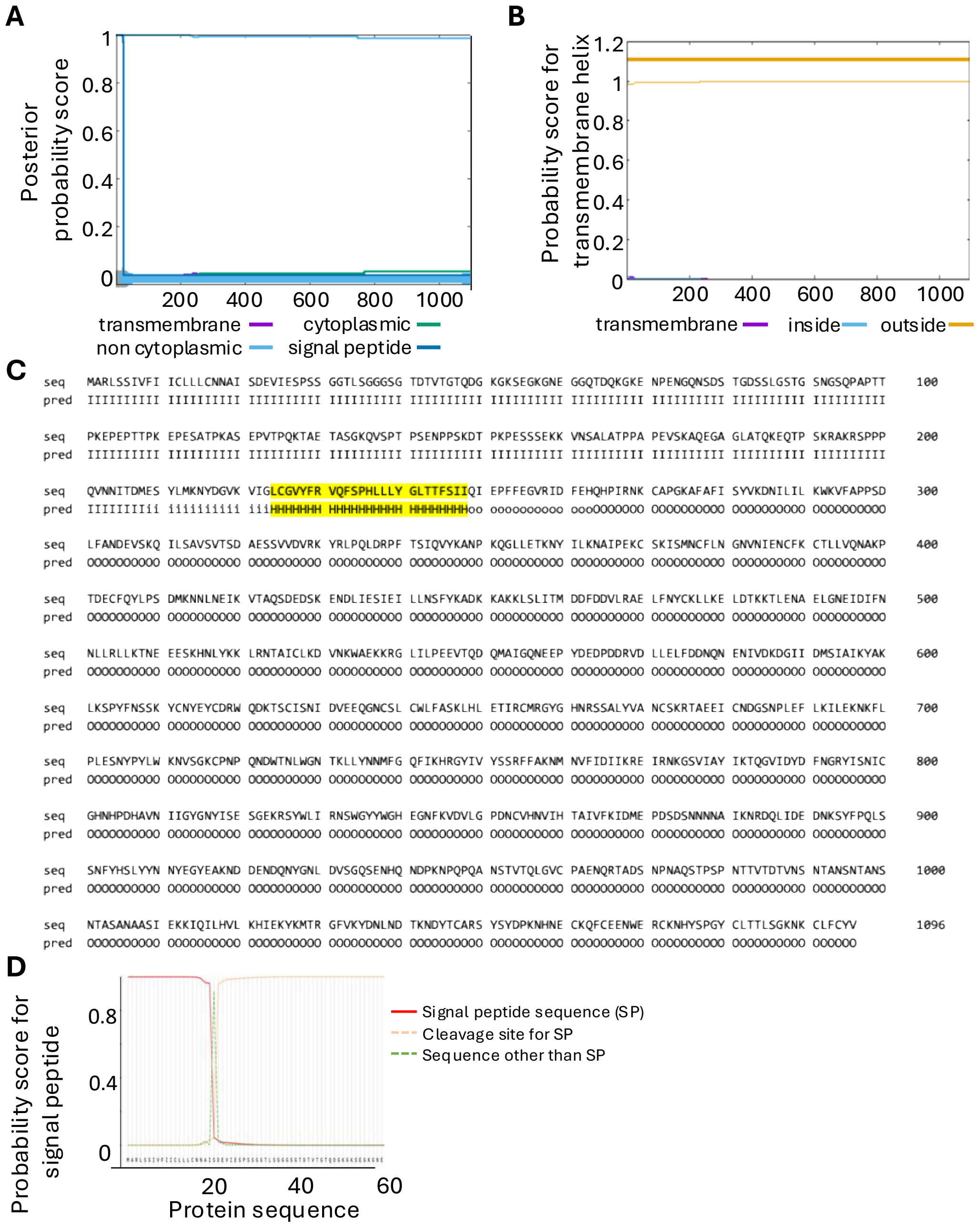

Figure S2: *In silico* predictions of functional motifs in PbSERA3. (A) Phobious posterior probability score showing non-cytoplasmic localisation of PbSERA3. (B) TMHMM score for the prediction of transmembrane motif and extracellular nature of PbSERA3. (C) HMMTOP prediction showing the transmembrane helix spanning the amino acids 224-248, indicated as the structural state 'H'. The structural states of amino acids inside loop are indicated as 'I", inside tail as "i", outside tail as "o" and outside loop as "O". (D) Prediction for signal peptide and its cleavage between amino acid residues 21 and 22 by SignalP-5.0.

Figure S3

A

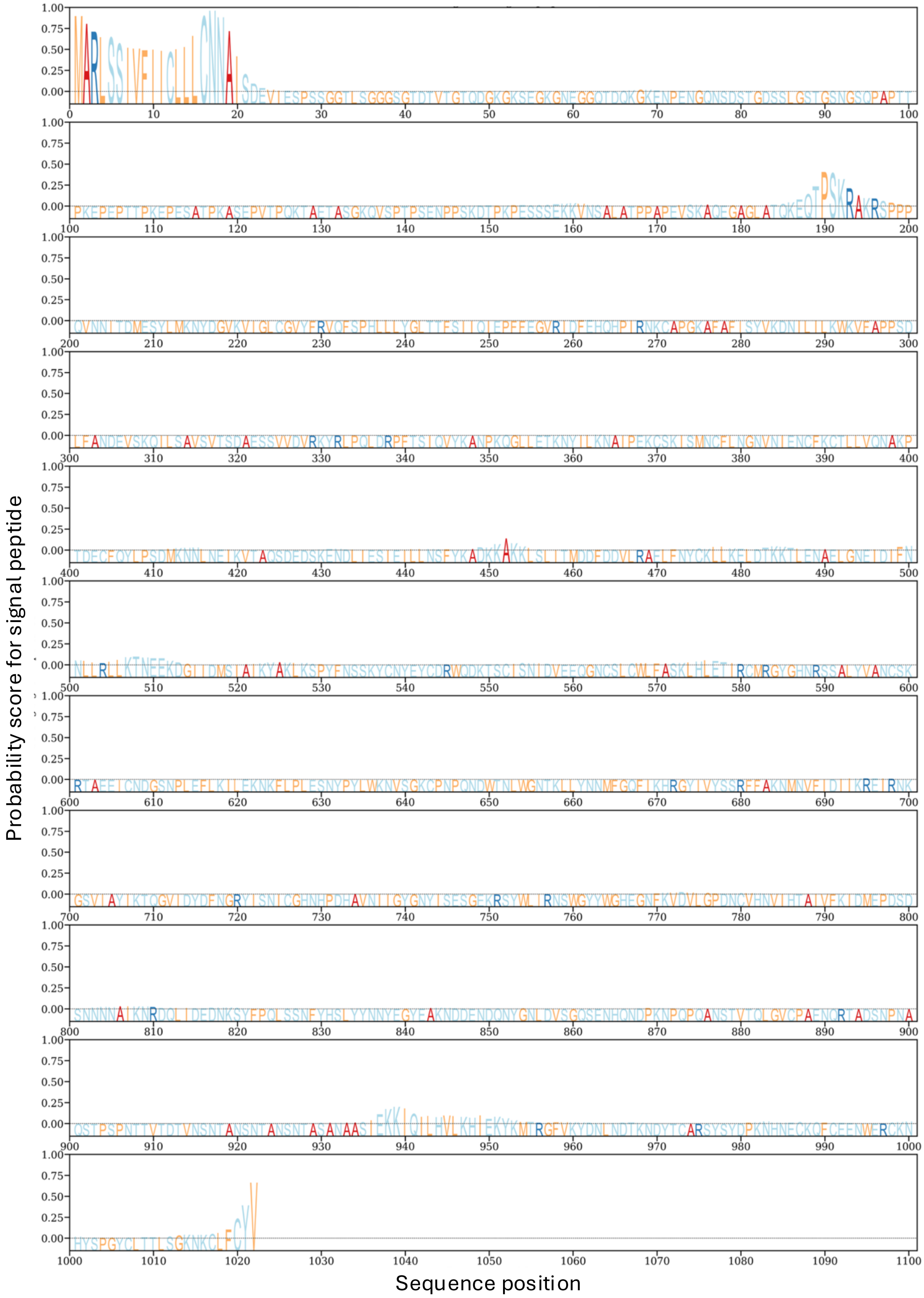

B

Localization	Cytoplasm	Nucleus	Extracellular	Cell membrane	Mitochondrion	plastid	Endoplasmic reticulum	Lysosome/ Vacuole	Golgi apparatus	Peroxisome
**Probability**	0.1881	0.1133	0.8700	0.5696	0.1.53	0.0177	0.2491	0.0892	0.2655	0.0388

Figure S3: (A) Signal peptide prediction of PbSERA3 using Deeploc 2.0. (B) Table showing the predicted scores for the localisation of PbSERA3 in different cellular compartments. According to the probability score generated by Deeploc 2.0, PbSERA3 is predicted to be extracellular.

Figure S4

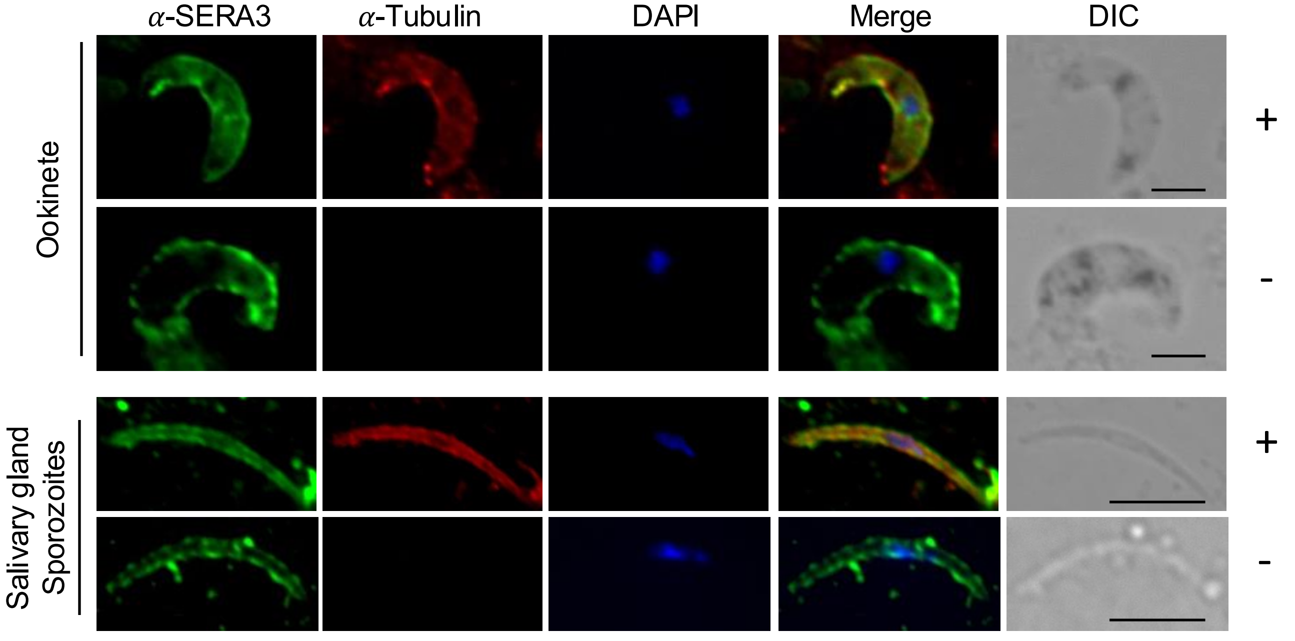

Figure S4: Fluorescent microscope images showing the localization of PbSERA3 in ookinete and SG sporozoites following staining with rabbit PbSERA3 antiserum under permeabilized (+) and non-permeabilized (-) conditions. Anti-Tubulin was used as a marker for ookinetes and sporozoites. Parasite nuclei were stained with DAPI (Scale bar - 5 µm).

Figure S5

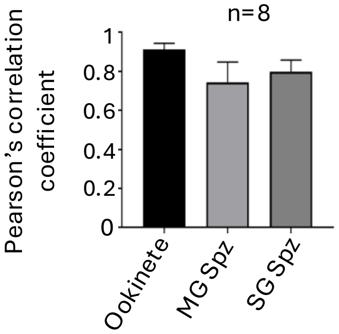

Figure S5: Pearson's correlation coefficient values for colocalization of PbSERA3 with p28 in ookinetes and with CSP in midgut and salivary gland sporozoites (n=8, per sample). The standard error of the mean (SEM) for ookinete, midgut sporozoite, and salivary gland sporozoite samples was ±0.01138, ±0.03664, and ±0.02091, respectively. MG Spz – midgut sporozoites and SG Spz – salivary gland sporozoites.

Figure S6

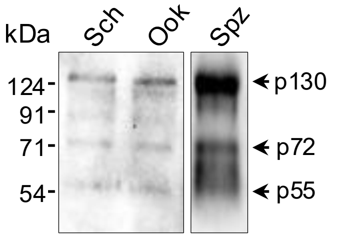

Figure S6: Western blot showing the precursor and mature forms of PbSERA3 in schizonts (Sch), ookinetes (Ook) and salivary gland sporozoite (Spz). The immunoreactivity was revealed with rabbit anti-PbSERA3 serum followed by a goat anti-rabbit HRP conjugated secondary antibody.

Figure S7

MARLSSIVFIICLLLCNNAISDEVIESPSSGGTLSGGGSGTDTVTGTQDGKGKSEGKGNEGGQTDQKGKENPENGQNSDSTGD
SSLGSTGSNGSQPAPTTPKEPEPTTPKEPESATPKASEPVTPQKTAETASGKQVSPTPSENPPSKDTPKPESSSEKKVNSALA
TPPAPEVSKAQEGAGLATQKEQTPSKRAKRSPPPQVNNITDMESYLMKNYDGVKVIGLCGVYFRVQFSPHLLLYGLTTFSIIQ
IEPFFEGVRIDFEHQHPIRNKCAPGKAFAFISYVKDNILILKWKVFAPPSDLFANDEVSKQILSAVSVTSDAESSVVDVRKYR
LPQLDRPFTSIQVYKANPKQGLLETKNYILKNAIPEKCSKISMNCFLNGNVNIENCFKCTLLVQNAKPTDECFQYLPSDMKNN
LNEIKVTAQSDEDSKENDLIESIEILLNSFYKADKKAKKLSLITMDDFDDVLRAELFNYCKLLKELDTKKTLENAELGNEIDI
FNNLLRLLKTNEEESKHNLYKKLRNTAICLKDVNKWAEKKRGLILPEEVTQDQMAIGQNEEPYDEDPDDRVDLLELFDDNQNE
NIVDKDGIIDMSIAIKYAKLKSPYFNSSKYCNYEYCDRWQDKTSCISNIDVEEQGNCSLCWLFASKLHLETIRCMRGYGHNRS
SALYVANCSKRTAEEICNDGSNPLEFLKILEKNKFLPLESNYPYLWKNVSGKCPNPQNDWTNLWGNTKLLYNNMFGQFIKHRG
YIVYSSRFFAKNMNVFIDIIKREIRNKGSVIAYIKTQGVIDYDFNGRYISNICGHNHPDHAVNIIGYGNYISESGEKRSYWLI
RNSWGYYWGHEGNFKVDVLGPDNCVHNVIHTAIVFKIDMEPDSDSNNNNAIKNRDQLIDEDNKSYFPQLSSNFYHSLYYNNYE
GYEAKNDDENDQNYGNLDVSGQSENHQNDPKNPQPQANSTVTQLGVCPAENQRTADSNPNAQSTPSPNTTVTDTVNSNTANSN
TANSNTASANAASIEKKIQILHVLKHIEKYKMTRGFVKYDNLNDTKNDYTCARSYSYDPKNHNECKQFCEENWERCKNHYSPG
YCLTTLSGKNKCLFCYV

Figure S7: Amino acid sequence of PbSERA3. The sequences of peptides used for immunisation are underlined.

Materials and methods

Experimental Animals

Female Swiss albino and C57BL/6 mice aged 6–8 weeks were procured from Hylasco Bio-Technology (India) Pvt. Ltd., Hyderabad, and housed at the animal facility of the University of Hyderabad. The animals were maintained at 22°C, with a relative humidity of 50–60%, and a 12-hour dark-light cycle. Animals were fed with a standard rodent diet provided ad libitum. An Institutional Animal Ethics Committee (IAEC) of the University of Hyderabad approved all the animal protocols conducted in this study.

Bioinformatic analysis of PbSERA3

The Phobius server (http://phobius.sbc.su.se/) was used to predict signal peptide and topology of PbSERA3 (1). Additionally, TMHMM 2.0 (https://services.healthtech.dtu.dk/services/TMHMM-2.0/) and HMMTOP (https://hmmtop.pbrg.hu/) were also used to predict transmembrane helices and topology of PbSERA3 (2, 3). The SignalP 5.0 (https://services.healthtech.dtu.dk/services/SignalP-5.0/) server and DeepLoc 2.0 server (https://services.healthtech.dtu.dk/services/DeepLoc-2.0/) were used to predict the presence of signal peptide and subcellular localisation of PbSERA3, respectively (4, 5).

Generation of pSTG-2FRT transfection plasmid and *Pbsera3*$^{FRT::GFP}$ plasmid

To generate the pSTG-2FRT plasmid, *3'utr* of *Pbtrap*, h*dhfr* cassette, and *FRT* fragments were amplified as a single PCR product from the plasmid p3'TRAP-flirte-hDHFR (6) using the primers P1/P2 (See **Table S1** for the details of primers and the sequences). The amplicon was digested with NotI and SalI and introduced into the NotI/XhoI restriction sites of pBC-GFP-hDHFR (7) to obtain pS1. GFP cassette with *5'* and *3'* regulatory sequences of *Pbhsp70* was released from pBC-GFP-hDHFR using the restriction sites ClaI and SmaI, and the sticky end of ClaI was blunted using T4 DNA Polymerase. pS1 was linearized with AflII, sticky ends were blunted with T4 DNA Polymerase, and ligated with the blunted GFP cassette to obtain pS2. To introduce the second FRT, the primers P3 and P4 were annealed *in vitro* and ligated into the SacII/NotI

restriction sites of pS2 to obtain the plasmid pSTG-2FRT (See Table **S1** for the details of primers and the sequences).

To generate *Pbsera3*$^{FRT::GFP}$ conditional knockout construct, the *5'* homology region (500 bp), promoter with *orf* (4.5 Kb) and *3'* homology regions (600 bp) of PbSERA3 were amplified from *P. berghei* genomic DNA using the primers P5/P6, P7/P8 and P9/P10 and introduced sequentially into SacII/SalI, XhoI/NotI and AscI/SacII restriction sites of pSTG-2FRT (See **Table S1** for the details of primers and the sequences), respectively. To successfully swap the *3'* regulatory sequence of *Pbsera3* with that of *TRAP*, the missing 12 bp *TRAP 3'UTR* in the pSTG-2FRT plasmid was added in the *Pbsera3 orf* after the stop codon.

Generation of PbSERA3$^{FRT::GFP}$ parasite line

The *Pbsera3*$^{FRT::GFP}$ conditional construct was linearized with SacII and transfected into *P. berghei* TRAP/FLPL parental line (where FLPL, a thermolabile variant of FLP recombinase driven by TRAP promoter, was introduced into a neutral P230p locus) following standard transfection procedure (8). The transfected parasites were selected by providing pyrimethamine in the drinking water of mice. The drug-resistant parasites were cloned by limiting dilution (9), and correct integration of the *Pbsera3*$^{FRT::GFP}$ conditional construct was confirmed by diagnostic PCRs from the genomic DNA of cloned parasite lines. The details of primers used for genotyping and the expected PCR product sizes are listed in **Table S1**. Clone 1 (cl1) of PbSERA3$^{FRT:GFP}$ parasite line was used for all the investigations unless specified.

Asexual propagation of PbSERA3$^{FRT:GFP}$ in mice and transmission to mosquitoes

To monitor the asexual propagation of blood stages, 1x10^6 infected RBCs from two clones (PbSERA3$^{FRT:GFP}$c1 and c2) obtained from independent transfections were introduced intravenously in C57BL/6 mice. Similar numbers of WT GFP parasites (7) infected in C57BL/6 mice served as controls. The parasitemia was monitored from days 4 to 7 by Giemsa staining (Sigma, Cat# 32884) of blood smears made from caudal vein puncture.

For mosquito transmission experiments, nearly 150–200 female *Anopheles* mosquitoes were fed on the anesthetized mice with circulating gametocytes of

PbSERA3$^{FRT:GFP}$ or wild-type parasites. The infected mosquitoes were maintained at 21°C with relative humidity of 75-80% and were supplemented with 5% sucrose.

Analysis of oocyst development inside the mosquito

To study the phenotype of the PbSERA3$^{FRT:GFP}$ line in mosquitoes, infected mosquito mid-guts were manually dissected using a dissection microscope (Lawerence & Mayo, Cat#NSZ-606) on day 14 post-infection. Oocysts were quantified, and sporulation patterns inside the oocysts were monitored. Oocyst sporozoite loads were determined following the disruption of dissected midguts using a plastic pestle and were counted using a hemocytometer.

Isolation of salivary gland sporozoites

Salivary glands were dissected from infected mosquitoes by dissection between days 18-21, post-infection, and were placed in a 1.5 ml tube containing RPMI-1640 media (Gibco, 11875093). The glands were disrupted using a plastic pestle, and the sporozoites were enumerated using a hemocytometer.

Determining the pre-patency

C57BL/6 mice were intravenously injected with $5x10^3$, $1x10^4$, $5x10^4$, or $1x10^5$ PbSERA3 cKO sporozoites. As a control, two groups of mice received $5x10^3$ and $1x10^4$ WT GFP sporozoites. Blood smears were made from all groups of mice from day 3 post-infection with sporozoites. Thin blood smears were made from infected mice by tail puncture. The slides were stained with Giemsa and observed under an oil immersion microscope by counting the parasites to determine the pre-patency.

HepG2 cell cultures for assessing *in vitro* EEF development

To investigate the developmental progression of the *PbSERA3 cKO* liver stages *in vitro*, parasite growth was assessed in HepG2 cells that were cultured in a 4-well plate (Thermo Scientific, Cat#144444) on collagen-coated coverslips at a density of $1x10^5$, one day prior to infection with sporozoites. On day 20-21post-infection of mosquitoes, the *PbSERA3 cKO* sporozoites were isolated, and approximately $2x10^4$ sporozoites were added to HepG2 monolayers and incubated at 37°C in a CO_2 incubator. Infected cultures

were fixed at 12 h, 36 h, and 62 h post infection with 4% paraformaldehyde (PFA) (Thermo Scientific, Cat#043368.9M) and processed for indirect immunofluorescence assay.

Quantification of parasite burden in liver by qRT PCR

Three groups of C57BL/6 mice (n=3/group) were intravenously infected with 1×10^4 sporozoites of either WT GFP or PbSERA3 cKO cl1 or cl2. Infected livers were isolated at 48 h and 70 h post-infection. The livers were homogenized in Trizol solution (Invitrogen, Cat# 15596026), and total RNA was isolated. Two µg of RNA was used to generate cDNA. qRT-PCR was performed using *P. berghei msp1* (See Table S1 for primer details). Mouse *gapdh* was used to normalize the Ct values of parasite genes following $2^{-\Delta\Delta Ct}$ method.

Indirect immunofluorescence Assay (IFA)

The fixed samples were permeabilised with cold acetone and methanol (1:3) and washed with PBS. All samples were blocked with 3% BSA and probed with rabbit anti-UIS4 antibody (kind gift from P. Sinnis, JHU) at 1:1000 dilution, which was used as a marker for PVM in hepatic stages (10) or rabbit PbSERA3 antibody at 1:500 dilution for its localization in the late liver stages. Primary antibodies were removed after 1 h incubation, washed three times with PBS, and incubated with AlexaFluor 594 conjugated chicken anti-rabbit secondary antibody (ThermoScientific, Cat#A21442) used at 1:1000 dilution. Nuclei were stained with DAPI (Sigma, Cat#D9564). The slides were mounted with coverslip using ProLong™ gold anti-fade reagent (ThermoScientific, Cat#P10144) and sealed with nail polish. For studying the localisation of PbSERA3 in sexual and sporozoite stages, the samples were spotted on the glass slide and fixed with 4% PFA in 1X PBS. Staining was performed in the presence and absence of permeabilization using 0.1% Triton X-100. Rabbit PbSERA3 antibody was used at 1:500 dilution. Anti-p28 (kind gift from A. Holder, Francis Crick Institute, London) and 3D11 antibodies were used as markers, respectively, for sexual stages (zygotes and ookinetes) and sporozoites at 1:1000 dilution. Mouse anti-Tubulin was used as a control for permeabilization experiments. The slides were washed three times with PBS, and incubated with AlexaFluor 488 conjugated goat anti-rabbit secondary antibody (ThermoScientific, Cat#A11008) and AlexaFluor 594 conjugated chicken anti-mouse secondary antibody (ThermoScientific, Cat#A21201) at 1:1000 dilution. Nuclei were stained with DAPI. The

slides were mounted with coverslip using ProLong™ gold anti-fade reagent and sealed with nail polish. For cytomere staining, the *in vitro* EEFs were fixed at 48 hr and permeabilized with cold acetone and methanol (1:3) and washed with PBS. The samples were blocked with 3% BSA and probed with mouse anti-MSP1 antibody (kind gift from A. Holder, Francis Crick Institute, London) at 1:1000 dilution and revealed with AlexaFluor 594 conjugated chicken anti-mouse secondary antibody at 1:1000 dilution. Nuclei were stained with DAPI. The slides were mounted with coverslip using ProLong™ gold anti-fade reagent and sealed with nail polish. The slides were visualized under a Nikon Eclipse upright fluorescence microscope, and images were captured and processed using the NIS-Elements AR software.

***In vitro* culture of ookinetes**

P. berghei gametocytes were enriched from asynchronous blood stage parasites as described earlier (11). Briefly, a Swiss albino mouse was intraperitoneally (i.p.) injected with 0.1 ml of 25 mg/ml phenylhydrazine (Sigma, Cat#114715). After 48 h of treatment, the mouse was infected i.p. with 10^7 iRBCs. The gametocytes were enriched by eliminating asexual blood stages using 25 mg/L sulfadiazine (Sigma, Cat#S8626) provided in drinking water to the mice during 48-72 h post-infection. The frequency of gametocytemia was estimated by Giemsa-stained tail blood smears. The gametocyte enriched blood was collected and diluted in ookinete culture medium [RPMI-1640, 20% FBS (Gibco, Cat#A5256701), 50 µM xanthurenic acid (Sigma, Cat#D120804), pH 8.2] and incubated at 21˚C for 16-24 h. Ookinetes were counted in a hemocytometer at 400x magnification under a light microscope. Ookinetes were collected by centrifugation at 1500 rpm for 8 min at room temperature.

Generation of PbSERA3 antisera

Peptides corresponding to amino acids 51-70 and 65-83 of PbSERA3 (Fig S7) were commercially synthesised from K.R. Instruments and Chemicals (KRIC), Kolkata, India. Each peptide of 250 μg was mixed with Freund's complete adjuvant (Sigma, Cat#F5881) and immunised intramuscularly, at multiple sites in a rabbit. Two subsequent booster doses were given at an interval of 14 days by mixing 125 μg of each peptide in Freund's

incomplete adjuvant (Sigma, Cat#F5506). The immune sera was collected 2 weeks after the second booster.

Western blotting

Parasite lysates were prepared from schizonts, ookinetes, and salivary gland sporozoites in PBS pH 7.2 (Gibco, Cat# 10010023). Following protein estimation by BCA method (ThermoScientific, Cat# 23225), the samples were denatured in SDS-sample buffer and resolved on a 10% SDS PAGE gel. The separated proteins were electroblotted onto a nitrocellulose membrane. The membrane was blocked with 3% BSA (Sigma, Cat# A9418) for one hour and three washes with PBS for 5 min each. The blot was probed with rabbit anti-SERA3 polyclonal serum at 1:5000 dilution in 3% BSA for one hour at room temperature. Anti-CSP monoclonal antibody 3D11 was used as a loading control to show the depletion of PbSERA3 in sporozoites. The immunoreactivity was revealed using anti-rabbit secondary antibody conjugated to HRP (Cell Signalling Technology, Cat# 7074) or anti-mouse secondary antibody conjugated to HRP (Cell Signalling Technology, Cat# 7076) and developed with chemiluminescent HRP substrate (Takara, Cat#T7103A) and imaged using Bio-Rad ChemiDoc MP imaging system.

Statistical analysis

GraphPad Prism v9.0 and above was used for statistical analysis. Statistical analyses were performed using the Mann-Whitney U test for the number of oocysts in mosquitoes, one-way ANOVA with Tukey's multiple comparison test for the quantification of midgut sporozoite numbers, salivary gland sporozoite numbers, *Pbmsp1* transcripts at 40 h and 70 h post-infection. An unpaired t-test was used to calculate the statistical differences for the cytomere quantification in EEFs at 48 h post-infection.

References

1. Kall L, Krogh A, Sonnhammer EL. 2007. Advantages of combined transmembrane topology and signal peptide prediction--the Phobius web server. Nucleic Acids Res 35:W429-32.
2. Krogh A, Larsson B, von Heijne G, Sonnhammer EL. 2001. Predicting transmembrane protein topology with a hidden Markov model: application to complete genomes. J Mol Biol 305:567-80.

3. Tusnady GE, Simon I. 2001. The HMMTOP transmembrane topology prediction server. Bioinformatics 17:849-50.
4. Almagro Armenteros JJ, Tsirigos KD, Sonderby CK, Petersen TN, Winther O, Brunak S, von Heijne G, Nielsen H. 2019. SignalP 5.0 improves signal peptide predictions using deep neural networks. Nat Biotechnol 37:420-423.
5. Thumuluri V, Almagro Armenteros JJ, Johansen AR, Nielsen H, Winther O. 2022. DeepLoc 2.0: multi-label subcellular localization prediction using protein language models. Nucleic Acids Res 50:W228-W234.
6. Lacroix C, Giovannini D, Combe A, Bargieri DY, Spath S, Panchal D, Tawk L, Thiberge S, Carvalho TG, Barale JC, Bhanot P, Menard R. 2011. FLP/FRT-mediated conditional mutagenesis in pre-erythrocytic stages of Plasmodium berghei. Nat Protoc 6:1412-28.
7. Al-Nihmi FM, Kolli SK, Reddy SR, Mastan BS, Togiri J, Maruthi M, Gupta R, Sijwali PS, Mishra S, Kumar KA. 2017. A Novel and Conserved Plasmodium Sporozoite Membrane Protein SPELD is Required for Maturation of Exo-erythrocytic Forms. Sci Rep 7:40407.
8. Janse CJ, Ramesar J, Waters AP. 2006. High-efficiency transfection and drug selection of genetically transformed blood stages of the rodent malaria parasite Plasmodium berghei. Nat Protoc 1:346-56.
9. Menard R, Janse C. 1997. Gene targeting in malaria parasites. Methods 13:148-57.
10. Mueller AK, Camargo N, Kaiser K, Andorfer C, Frevert U, Matuschewski K, Kappe SH. 2005. Plasmodium liver stage developmental arrest by depletion of a protein at the parasite-host interface. Proc Natl Acad Sci U S A 102:3022-7.
11. Beetsma AL, van de Wiel TJ, Sauerwein RW, Eling WM. 1998. Plasmodium berghei ANKA: purification of large numbers of infectious gametocytes. Exp Parasitol 88:69-72.

Table S1: List of primers

Primers used to generate pSTG-2FRT-GFP and *Pbsera3*$^{FRT::GFP}$ plasmid

DNA Construct	Primer ID	Sequence	Enzymes	Product (bp)	Primer description
pSTG-2FRT plasmid	P1	ATGCGGCCGCGATATCTATATATCTAGATGATTATTCTTATGTTAC	NotI	2280	*Pbtrap 3'utr* forward
	P2	GAGTCGACCTGCAGGCGCTTAAGGAAGTTCCT	SalI		*FRT* reverse
	P3	GGTGGCGGCCGTCGACGAAGTTCCTATTCTCTAGAAAGTATAGGAACTTCGAATTCTCGAGGCGCCGCATGC			*FRT* oligo forward
	P4	GGCCGCATGCGGCGCCTCGAGAATTCGAAGTTCCTATACTTTCTAGAGAATAGGAACTTCGTCGACGGCCGCCACCGC			*FRT* oligo reverse
Pbsera3$^{FRT:GFP}$ construct	P5	CGACCGCGGTATACGTATGTGCAATTAATA	SacII	500	*Pbsera3 5' HR* forward
	P6	GGCGTCGACATAAACTTGTTATAGTACAATGT	SalI		*Pbsera3 5' HR* reverse
	P7	GCCCTCGAGTGTTACACAACTAAGGAGAAAA	XhoI	4556	*Pbsera3 promoter* forward
	P8	GTAGCGGCCGCATATGTTTATTAAAATTATACATAACAGAAGAGACA	NotI		*Pbsera3 orf* reverse
	P9	GACGGCGCGCCTTTTGAGGTGTGTGCAAATAA	AscI	605	*Pbsera3 3' HR* forward
	P10	CTGCCGCGGTTCCTCTTCAATTGTGTATTAC	SacII		*Pbsera3 3' HR* reverse

Primers used for *Pbsera3*$^{FRT:GFP}$ integration PCRs

Parasite line	Primer ID	Sequence	Product (bp)	Primer description
Pbsera3$^{FRT:GFP}$ integration confirmations	P11	ATGTAACTTTTGCGCTGTATA	597	*Pbsera3 5'* int forward
	P12	TACTTTCTAGAGAATAGGAACT		*Pbsera3 5'* int reverse
	P13	TACACTTTATGCTTCCGGCT	695	*Pbhsp70 5'utr* forward
	P14	TATTTTTATCCTTTTTCGTCA		*Pbsera3 3'* int reverse
	P15	ACTCCACGTGTTAAAGCATATCG	755	*Pbsera3 orf* forward
	P16	TATATGAACTTAAAGACGAATA		*Pbtrap 3'utr* reverse

Primers used for the confirmation of excision in *Pbsera3$^{FRT:GFP}$* parasites

Parasite line	Primer ID	Sequence	Product (bp)	Primer description
Pbsera3$^{FRT:GFP}$ non-excised	P17	GTTGGACTTGATTTTTAAAATG	660	*Pbdhfr 3' utr* forward
	P18	TTTGCAAGCATAATTAAGATGA		*Pbhsp70 3'utr* reverse
Pbsera3$^{FRT:GFP}$ excised	P19	TTACACACATAAAATGGCTAGTA	900	*Pbsera3 5' HR* forward
	P18	TTTGCAAGCATAATTAAGATGA		*Pbhsp70 3'utr* reverse

Primers used for the qRT PCR

Parasite line	Primer ID	Sequence	Product (bp)	Primer description
Pbmsp1 qRT PCR	P20	AATGCTGGATGTTTTAGATATGA	129	*Pbmsp1* qPCR forward
	P21	ATCACATCCACCATTGTTGTTTCC		*Pbmsp1* qPCR reverse
Mouse gapdh qRT PCR	P22	CCTCAACTACATGGTCTACAT	122	Mouse *gapdh* qPCR forward
	P23	GCTCCTGGAAGATGGTGATG		Mouse *gapdh* qPCR forward

orf - open reading frame, utr - untranslated region, HR – homologous region, FRT-FLP recognition target, and int - integration
